# Laboratory-based and office-based Globorisk scores to predict 10-year risk of cardiovascular diseases among Iranians: results from the Fasa PERSIAN cohort

**DOI:** 10.1186/s12874-022-01791-7

**Published:** 2022-11-26

**Authors:** Leila Jahangiry, Azizallah Dehghan, Mojtaba Farjam, Dagfinn Aune, Fatemeh Rezaei

**Affiliations:** 1 Road Traffic Injury Research Center, Tabriz Health Services Management Research Center, Tabriz University of Medical Sciences, Tabriz, Iran; 2 Health Education and Health Promotion Department, School of Health, Medical Education Research Center, Health Management and Safety Promotion Research Institute, Tabriz University of Medical Sciences, Tabriz, Iran; 3 Noncommunicable Diseases Research Center, Fasa University of Medical Sciences, Fasa, Iran; 4 Department of Epidemiology and Biostatistics, School of Public Health, Imperial College London, London, UK; 5 Department of Nutrition, Bjørknes University College, Oslo, Norway; 6 Research Center for Social Determinants of Health, Jahrom University of Medical Sciences, Jahrom, Iran

**Keywords:** Laboratory-based, Office-based, Globorisk, Cardiovascular disease, Risk prediction

## Abstract

**Background:**

Globorisk is a novel risk prediction model for predicting cardiovascular disease (CVD). Globorisk is a country-specific risk prediction model that determines CVD risk for all countries. This model has two versions; laboratory-based and office-based. This study aimed to determine the agreement between laboratory-based and office-based models in a large sample of the general population.

**Methods:**

Baseline data from the Fasa cohort study was used for the current study. In total, 6810 participants ≥ 40 years without any history of cardiovascular disease or stroke were included in the study. To determine the laboratory-based risk model, factors include age, sex, current smoking status, history of diabetes, systolic blood pressure (SBP), and total cholesterol. To estimate the office-based risk model, factors were age, sex, current smoking status, SBP, and body mass index (BMI). Kappa statistics was used to distinguish the agreement between grouped scores in these two models. Additionally, correlation coefficients and scatter plots were used to determine the linear correlation between the two models.

**Results:**

In this study 46.53% of the participants were men. The mean age (SD) of participants was 51.08 (7.88) years. Agreements between the two models were moderate and substantial in all women and all men, respectively. The agreement between the two CVD risk groups was 90.15% (kappa = 0.717) in all men, 92.94% (kappa = 0.571) among men aged < 60 years and 77.60% (kappa = 0.645) in men aged ≥ 60 years. The agreement between the two CVD risk groups was 86.68% (kappa = 0.572) among all women, 93.96% (kappa = 0.274) among women aged < 60 years and 62.46% (kappa = 0.422) among women aged ≥ 60 years. A very strong positive correlation (r = 0.94) was found between the two risk scores in all men, and it was similar among men aged < 60 years (r = 0.84) and men aged > 60 years (r = 0.94). Among all women, there was a very strong positive correlation (r = 0.87), and the strong positive correlation remained among < 60 years old (r = 0.76) and women > 60 years old (r = 0.76).

**Conclusion:**

The Globorisk office-based model which is easier to use as it does not require blood testing can determine the risk groups in this population. The Globorisk office-based model may be used for CVD risk screening in low-middle income countries where resources are limited.

**Supplementary Information:**

The online version contains supplementary material available at 10.1186/s12874-022-01791-7.

## Background

Cardiovascular diseases (CVDs) are the leading cause of death and disability worldwide [1]. More than three-quarters of CVD-related deaths occur in low and middle income countries (LMICs) [2]. An estimation shows that 17.9 million people died of CVD in 2016 and accounted for 31% of all global deaths. Evidence shows that 85% of these deaths were due to heart attacks and strokes. More than four-fifths of premature deaths [3] (deaths before age 70) are due to non-communicable diseases in middle income countries (MICs), and more than one-third are due to CVD. The incidence of early CVD in the Iranian population is 5.06 per 1000 person-years [4].

Although the standardized age prevalence of CVD in high-income countries (HICs) is declining, this declining trend is not as evident for most LMICs, where current rates are more than 9,000 common cases per 100,000. There has been a significant decline in age-standardized CVD mortality rates in all HICs, while similar changes were not observed in most North Africa and Middle East countries, such as Iran [2, 5].

Two population based and individual based strategies have been recommended for prevention of CVD. Both strategies require CVD risk measurement to shift the risk distribution to lower levels and treat high-risk individuals [6]. Prevention of CVD requires early diagnosis of people who are at higher risk to identify effective dietary, lifestyle, or medication interventions for them. Despite the high incidence of CVD in LMIC, most knowledge about risk prediction has been obtained from cohort studies in HIC. Over the past two decades, many predictive models have been developed that mathematically combine multiple predictors to estimate the risk of developing CVD [7, 8]. The most commonly used method is to use a total risk score to predict cardiovascular risk, such as the Framingham Risk Score for Americans and the Systematic Coronary Risk Evaluation (SCORE) for Europeans. However, these methods have been developed for European and American countries [7, 8].

No country-specific CVD risk prediction tools has been developed in Iran and the developed tools in other countries or international tools are therefore used in studies from LMICs as well. Globorisk was invented for estimation of CVD risk score in 2015 [6]. Globorisk is one of the newly developed CVD risk score calculators which uses a country-specific model as compared to others and predicts the risk of heart attack or stroke in healthy people (those who have not yet had a heart attack or stroke) across countries [9]. Previously, CVD risk assessment instruments were based on data from high-income countries. However, CVD-related deaths are higher among low-income countries. Specific factors to a country such as age, gender, smoking, diabetes, SBP, and cholesterol are needed to estimate Globorisk CVD risk score [10]. It seems that using Globorisk CVD risk calculator is a great step forward for global CVD prevention.

Globorisk has two versions, the Globorisk laboratory-based and office-based CVD risk calculator [10]. In the Globorisk office-based, total cholesterol, and diabetes replaced with BMI for CVD risk calculation because the association between BMI and both cholesterol and diabetes is very strong, and also because excess weight has a direct effect on these physiological traits [6, 10, 11]. In addition, body weight, blood sugar, and serum cholesterol increase with improper diet and physical inactivity [6].

In LMICs, laboratory tests and facilities may not be available to everyone at primary care centers, and people may not be able to pay for laboratory tests, so if a person does not have a blood test, the office-based version of the Globorisk can be useful to determine CVD risk score using BMI instead of blood sugar and serum cholesterol. Numerous studies have examined the agreement between CVD risk prediction models. In some countries, BMI-based and cholesterol-based versions of the Framingham risk score and WHO risk charts have been compared. The studies have been estimated moderate and good agreements [12–15]. Framingham risk score, WHO risk assessment tool, and Systematic Coronary Risk Evaluation (SCORE) have been compared in Iran [16–20], there is currently no studies comparing Globorisk assessment tools. It is necessary to examine the agreement between the laboratory-based and office based versions of Globorisk. Due to the high prevalence of CVD in Iran and the lack of laboratory facilities in some primary health centers, this study aimed to compare and examining the agreement between Globorisk laboratory-based and office-based models in a large Iranian population.

## Methods

**Fig. 1:**
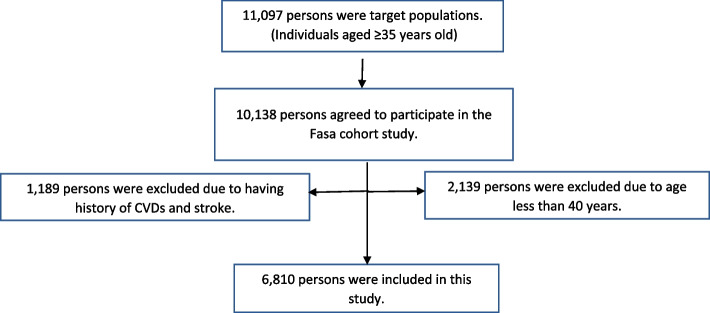
Study flow chart

This study was performed cross-sectionally using the baseline data of the Fasa cohort study from southwest of Iran. More details of the study protocol have been documented elsewhere [21]. Briefly, the target population of the Fasa cohort study was 11,097 individuals aged ≥ 35 years old and 10,138 individuals agreed to participate in the study. As the Globorisk equations have been developed for people aged 40–80 years old [6, 22], 2139 persons with age less than 40 years old were excluded. Also, we excluded 1189 persons due to having history of CVDs and stroke. Finally, 6810 persons were eligible for the study (Fig. 1).

The data collection was done from 2015 to 2016. Demographic characteristics (age and sex) of participants, lifestyle factors, and disease history were collected by trained interviewers. Also, information on anthropometric indices (measured height and weight), the current status of smoking (yes/ no), alcohol intake (yes/ no), and medical history including diabetes, hypertension, and CVDs history were measured. Fasting blood samples were collected for in order to measure high-density lipoprotein (HDL), low-density lipoprotein (LDL), cholesterol, and triglycerides.

### CVD risk estimation

The 10-year risk of fatal and non-fatal CVD was calculated using Globorisk laboratory-based and office-based models. The laboratory-based model was estimated by using age, sex, SBP, current smoking status, diabetes, and total cholesterol (mmol/L). The office-based model was calculated using age, sex, SBP, current smoking, and BMI (kg/m2).

In the office-based Globorisk CVD risk score, diabetes and total cholesterol are replaced with BMI as BMI is known as a proxy for raised blood sugar and serum cholesterol [9, 10]. SBP of participants was measured twice using a mercury sphygmomanometer after resting then their average was recorded in mmHg. Overnight fasting (10–14 h) was recommended for participants when they were invited to blood sampling. Diabetes was defined as fasting blood sugar (≥ 126 mg/dL) or having a history of diabetes. BMI was calculated as weight (kg) divided by the height square (meter). Current smoking status was obtained by yes/no questions.

### Ethical considerations

This study was approved by the Ethics Committee of Jahrom University of Medical Sciences (IR.JUMS.REC.1400.071). Data were obtained from Farjam et al. study [21].

### Statistical analysis

Categorical and continuous variables were reported as percentages and means (standard deviations, SDs). Chi-square and t-test were used for categorical and continuous variables, respectively. For the Globorisk equations estimation for Iranian population, according to Ueda et al. study [10], the high-risk group was considered to be ≥ 20%. Therefore, the Globorisk scores were categorized into three groups including low (< 10%), moderate (10% to < 20%), and high (≥ 20%) ≥ 20%. The Kappa statistic was used to assess the agreement between laboratory-based and office-based models. The correlation coefficients and scatter plots were used to determination linear correlation. Kappa statistics and correlation coefficients between the laboratory-based and office-based models were calculated stratified by gender and age groups (< 60 and ≥ 60 years) to determine the agreement and correlation between laboratory-based and office-based models in these subgroups. Scatter plots were used for reporting continuous risk scores. The correlation between the two models of individual CVD risk scores was assessed by scatter plots. The correlation coefficient was classified as very weak (r = 0.00–0.19), week (r = 0.20–0.39), moderate (r = 0.40–0.59), strong (r = 0.60–0.79), and very strong (r = 0.80–1.00) [23]. For categorical risk scores in the Globorisk CVD risk model, we categorized the predicted risk into three groups including low-, moderate- and high-risk groups. Kappa statistics were used for assessing the agreement between the categorized risk of the laboratory-based and office-based models. The Kappa agreement was classified as odds (kappa < 0), fair (kappa = 0.21– 0.40), moderate (kappa = 0.41–0.60), substantial (kappa = 0.61–0.80), and almost complete (kappa = 0.81–0.99) [24].

Statistical analyses were performed with Statistical Package for Social Science (IBM SPSS Statistics for Windows, Version 23.0. Armonk, NY: IBM Corp) and Stata Statistical Software (Stata 14 for windows, Stata Corp., College Station, TX, USA). P-values < 0.05 were considered as statistically significant.

## Results

In this study 46.53% of the participants were men. The mean age (SD) of the participants was 51.08 (7.88), and 17.42% was aged ≥ 60 years. The prevalence of diabetes and hypertension was higher in women than men. The prevalence of smoking was higher in men than women. The mean (SD) of BMI, HDL, LDL, Cholesterol, SBP, and DBP were higher in women than men. Mean scores (SD) for office-based Globorisk CVD risk score was higher than laboratory-based CVD risk score (6.26 (6.73) vs. 6.18 (7.22)). Also, the mean score of office-based and laboratory-based CVD risk scores in men were higher than in women (Table 1).

**Table 1:** The participants’ characteristics

Variables	Total (*n* = 6810) N (%)	Men (*n* = 3169) N (%)	Women (*n* = 3641) N (%)	*P*-value
Age range (years)				
< 60	5624 (82.58)	2593 (81.82)	3031 (83.25)	0.123*
≥ 60	1186 (17.42)	576 (18.18)	610 (16.75)	
Marital status				
Married	6071 (89.15)	3116(98.33)	2955(81.18)	< 0.001*
Other	739 (10.85)	53(1.67)	686 (18.84)	
Education level				
Illiterate	3573 (52.47)	1247 (39.35)	2326 (63.88)	< 0.001*
≤diploma	3138 (46.08)	1838 (58)	1300 (35.70)	
University	99 (1.45)	84 (2.65)	15 (0.41)	
Smoking (now)				
No	5456 (80.12)	1901 (59.99)	3555 (97.64)	< 0.001*
Yes	1354 (19.88)	1268 (40.01)	86 (2.36)	
Hypertension				
No	5516 (81.00)	2840 (89.62)	2676 (73.50)	< 0.001*
Yes	1294 (19.00)	329 (10.38)	965 (26.50)	
Diabetes				
No	5937 (87.18)	2917 (92.05)	3020 (82.94)	< 0.001*
Yes	873 (12.82)	252 (7.95)	621 (17.06)	
DBP (Mean mmHg ± SD)	75.04 ± 11.84	74.58 ± 11.72	75.44 ± 11.94	< 0.001**
SBP (Mean mmHg ± SD)	112.44 ± 18.44	111.29 ± 17.59	113.43 ± 19.11	< 0.001**
HDL (Mean mmol/l ± SD)	1.32 ± 0.41	1.23 ± 0.37	1.41 ± 0.42	< 0.001**
Chol (Mean mmol/l ± SD)	4.86 ± 1	4.67 ± 0.94	5.03 ± 1.02	< 0.001**
BMI (kg/m^2^)	25.54 ± 4.83	24.16 ± 4.46	26.74 ± 4.84	< 0.001**
Laboratory-based CVDs risk score (10- year,%), (Mean ± SD)	6.18 ± 7.22	7 ± 6.71	5.47 ± 7.57	< 0.001**
Office-based CVD risk score (10- year,%), (Mean ± SD)	6.26 ± 6.73	7.45 ± 6.81	5.22 ± 6.49	< 0.001**

*DBP* Diastolic blood pressure, *SBP* Systolic blood pressure, *HDL* High density lipoprotein, *Chol* Cholesterol

*chi-square test, **t-test

**Fig. 2:**
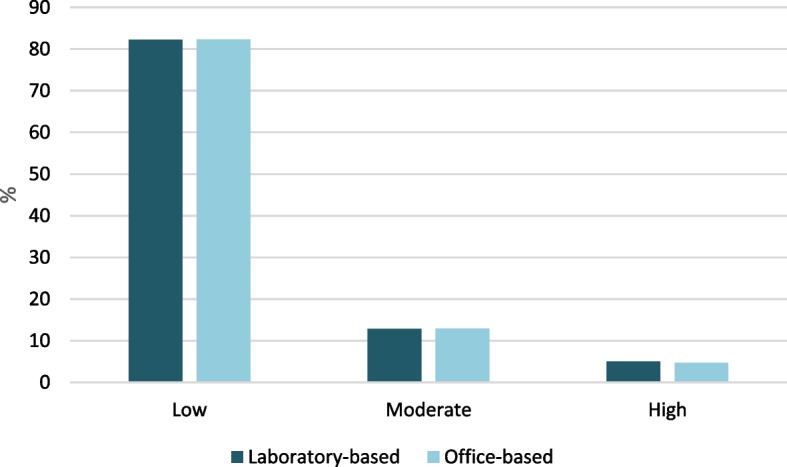
The percentage of the 10-years Globorisk CVD risks classified according to laboratory-based and office-based models

The risk classification of the laboratory-based and office-based Globorisk models are shown in Fig. 2. The 10-years risk classification of laboratory-based and office-based models were very similar. So that, 5% and 4.7% of participants were high risk in the laboratory-based and office-based models, respectively.

### Categorical agreement

The agreements between the laboratory-based and office-based Globorisk CVD risk scores according to the grouped risk (low, moderate, and high) for men and women are shown in Tables 2 and 3. The agreement between two CVD risk score were 90.15% (kappa = 0.717) in all men and 168 participants in office-based versus 162 participants in laboratory-based were in high risk group. The agreement was 92.94% (kappa = 0.571) among men aged < 60 years and 22 participants was in high risk group in laboratory-based model in comparison to 11 participants in the office-based model. Also, the agreement was 77.60% (kappa = 0.645) in men aged ≥ 60 years. According to the results 157 men aged ≥ 60 in the office-based and 140 men aged ≥ 60 in laboratory-based were in high risk group.

**Table 2:** Agreement between the laboratory-based and office-based risk scores according to the grouped risk in men

Office-based risk category	laboratory-based risk category				Agreement (%)	Kappa (SE)
	Low	Moderate	High	Total		
All men						
Low	2386	92	4	2482	90.15	0.717(0.014)
Moderate	142	345	32	519		
High	0	42	126	168		
Total	2528	479	162	3169		
< 60 years old						
Low	2277	76	4	2357	92.94	0.571(0.027)
Moderate	86	127	12	225		
High	0	5	6	11		
Total	2363	208	22	2593		
≥ 60 years old						
Low	109	16	0	125	77.60	0.645 (0.028)
Moderate	56	218	20	294		
High	0	37	120	157		
Total	165	271	140	576		

**Table 3:** Agreement between the laboratory-based and office-based risk scores according to the grouped risk in women

Office-based risk category	laboratory-based risk category				Agreement (%)	Kappa (SE)
	Low	Moderate	High	Total		
All women						
Low	2953	163	9	3125		
Moderate	114	176	72	362	88.68	0.572(0.017)
High	0	54	100	154		
Total	3067	393	181	3641		
< 60 years old						
Low	2817	120	6	2943		
Moderate	41	24	13	78	93.96	0.274(0.034)
High	0	3	7	10		
Total	2858	147	26	3031		
≥ 60 years old						
Low	136	43	3	182		
Moderate	73	152	59	284	62.46	0.422(0.031)
High	0	51	93	144		
Total	209	246	155	610		

The agreement between two CVD risk scores was 88.68% (kappa = 0.572) for women. Of the women, 181 participants in laboratory-based and 154 participants in office-based risk group were in the high risk group. The agreement was 93.96% (kappa = 0.274) among women aged < 60 years and 10 participants in the office-based model in comparison to 26 participants in the laboratory-based model were at high risk. Also, the agreement was 62.46% (kappa = 0.422) in women aged ≥ 60 years, 155 women in the laboratory-based and 144 women in office-based group were at high risk.

### Correlation coefficients

**Fig. 3:**
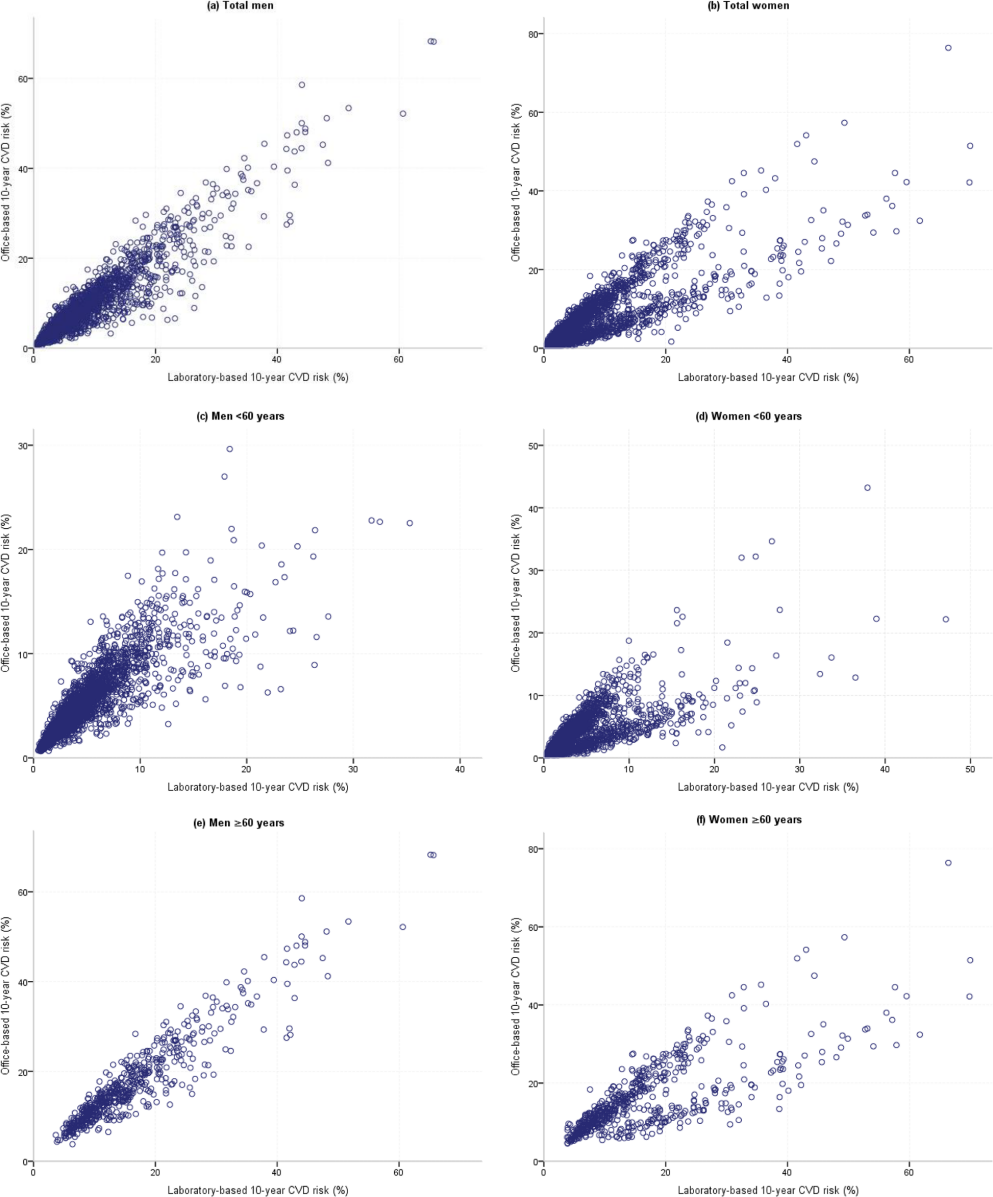
linear relationship between Globorisk laboratory-based and office-based models

The scatter plot shows a linear relationship between the two models in Fig. 3. The Pearson’s correlation coefficient is shown in Table 4. There was a very strong positive correlation between two models (r = 0.94) in all men and also in men < 60 years (r = 0.84) and men ≥ 60 years old (r = 0.94) with a highly significant *P*-value (*P* < 0.001). Also, the Pearson’s correlation coefficient was 0.87 for all women and 0.76 for women < 60 and ≥ 60 years old with a highly significant *P*-value (*P* < 0.001).

**Table 4:** Pearson’s correlation coefficient of the predicted individual-level risk of cardiovascular disease using the office-based with laboratory-based model

	*N*	r^a^ (95% CI)	*P*-value	Comment^b^
Men				
All men	3169	0.94 (0.94, 0.94)	< 0.001	Very strong positive
< 60 years old	2593	0.84 (0.83, 0.85)	< 0.001	Very strong positive
≥ 60 years old	576	0.94 (0.93, 0.95)	< 0.001	Very strong positive
women				
All women	3641	0.87 (0.86, 0.88)	< 0.001	Very strong positive
< 60 years old	3031	0.76 (0.74, 0.77)	< 0.001	strong positive
≥ 60 years old	610	0.76 (0.72, 0.79)	< 0.001	strong positive

^a^Correlation coefficient

^b^00-0.19 “very weak”, 0.20-0.39 “weak”, 0.40-0.59 “moderate”, 0.60-0.79 “strong”, 0.80 − 1.0 “very strong”

## Discussion

In this study we compared laboratory-based and office-based 10-years Globorisk CVD scores. The results showed that there was a substantial agreement among office-based and laboratory-based. 10-year Globorisk CVD risk scores in the classification of risks indicated that there was good agreement for men and women. Also, there was a very strong positive correlation between two risk scores for men and women.

Some studies have evaluated the agreement between different models that predict the risk of cardiovascular disease [25, 26]. Jones et al. published the agreement between the two Framingham risk scores [12]. Also, Rezaei et al. showed that the non-laboratory-based Framingham risk score has good agreement with the laboratory-based model [17].

In this study, firstly, the risk scores were categorized into low (< 10%), moderate (10% to < 20%), and high (**≥** 20%) risk groups. Then, kappa statistics were used for evaluating the agreement between the categorized risks of two models. The results showed that, the agreement was better in all men than in all women. The agreement was substantial for all men (kappa = 0.717) and moderate for all women (kappa = 0.572). On the other hand, we observed the considerable agreement in men aged < 60 years (kappa = 0.571) and > 60 years (kappa = 0.645). The agreement was fair (kappa = 0.274) for women aged < 60 years and moderate (kappa = 0.422) for women **≥** 60 years. A similar study reported that there was high agreement between office-based and laboratory-based Framingham models for men (92.2%) and women (93.4%). In this study, percent agreement reported was in the range between 91.9 and 95.7% and 94.2–95.1% across the laboratory-based scores for men and women, respectively. Additionally, for a threshold of 10-year CHD risk > 20%, the corresponding agreement ranged from 94.9 to 96.5% and 96.6–97.9% for men and women, respectively [27]. Rezaei and et al. by using WHO risk model showed that the non-laboratory-based risk prediction model classifies individuals almost identically to the laboratory-based model [18]. The results of a study showed that BMI is a good proxy blood sugar, and serum cholesterol [9, 10], which could replace these variables in settings where cost may be more prohibitive for using the laboratory-based model.

In our study, the 10-years CVD risk groups was very similar in the laboratory-based and office-based models. A small number of participants in both models were in the high-risk group. This means that 5% in the laboratory-based model and 4.7% in the office-based model were in the high-risk group. The risk of moderate to high was 17.8% for the laboratory-based model and 17.6% for office-based model. Another study from Malaysia showed that the risk score **≥** 20% was higher than our study indicating that high risk groups in women and men were 7.9% and 19%, respectively [9]. One of the reasons for the difference between Iran and Malaysia’s risk scores is related to the nature of Globorisk CVD risk score. Globorisk is a CVD risk score calculator which is country-specific and the differences between population features of the countries probably may have an effect on their calculated risk scores. Another potential reason for differences in the Globorisk score would be attributed to the Fasa cohort population, where most of the people live in villages or small towns and may have a relatively healthy lifestyle when compared to the people living in urban areas. It is necessary to mention for the Globorisk equations, risk grouping has not been specified as like as other CVDs risk prediction tools. In this study, the high-risk group was considered to be ≥ 20%.

In the present study, we used correlation coefficients and scatter plots to show correlation between office-based and laboratory-based models. The result showed that there was a very strong positive correlation (r = 0.94) between two risk scores in all men. Also, there was a very strong positive correlation in men aged < 60 years and men aged ≥ 60 years. In all women, there was a very strong positive correlation (r = 0.87), but the correlations were strong positive in women < 60 years and women ≥ 60 years. Pandya and et al. evaluated the correlation between the laboratory-based and non-laboratory-based Globorisk models to predict the risk of CVD by using Framingham model showed that the correlation between laboratory-based and non-laboratory-based in men and women were 0.957 and 0.946, respectively [27].

Recently, Globorisk was developed and depicted different models for each country. The Globorisk calculator for CVD risk that has been developed for 182 countries may be an important tool for the primary prevention of CVD globally [9]. Globorisk has two risk calculators including office-based and laboratory-based risk calculator for prediction of 10-year risk of CVD. These calculations are easy to use for CVD risk prediction by primary health providers. In LMICs, especially in primary health centers where people may not have access to a laboratory facilities for testing, or where people cannot afford testing, the non-laboratory or office-based model can be used as an easy to implement and cheaper alternative to laboratory tests to determine 10-year CVD risk. The Globorisk calculator has public health importance to distinguish people at high risk from those at low or medium risk and could help reduce costly health care through primary prevention.

### Study strengths and limitations

The main strength of the present study is the large sample size and the use of carefully collected data from a population-based study. Since, the study was conducted in a small city from southwest of Iran, the findings cannot be generalizable for the Iranian general population. This study was the first research comparing two laboratory-based and office-based models using the Globorisk risk score in a large population. However, this study is a cross-sectional study, to confirm the findings of this study, longitudinal studies with 10-years follow-up should be performed.

## Conclusion

The present study results provide key evidence that the correlation coefficients of laboratory-based and office-based 10-years Globorisk CVD risk scores were strong for all age groups and in both men and women, and there was substantial agreement between office-based and laboratory-based Globorisk scores when the scores were classified, and also when categorized into three groups. A low-cost model that is easy and without the need for blood testing can determine low-risk, moderate, and high-risk individuals to a similar extent as laboratory-based models. Therefore, the use of this office-based tool could be especially useful in LMICs where resources are limited.

## Supplementary Information

**Additional fale 1**.

## Data Availability

All data generated or analyzed during this study are available from supplementary File 1.
