# Correction: Laboratory-based and office-based Globorisk scores to predict 10-year risk of cardiovascular diseases among Iranians: results from the Fasa PERSIAN cohort

**DOI:** 10.1186/s12874-022-01808-1

**Published:** 2022-12-07

**Authors:** Leila Jahangiry, Azizallah Dehghan, Mojtaba Farjam, Dagfinn Aune, Fatemeh Rezaei

**Affiliations:** 1 Road Traffic Injury Research Center, Tabriz Health Services Management Research Center, Tabriz University of Medical Sciences, Tabriz, Iran; 2 Health Education and Health Promotion Department, School of Health, Medical Education Research Center, Health Management and Safety Promotion Research Institute, Tabriz University of Medical Sciences, Tabriz, Iran; 3 Noncommunicable Diseases Research Center, Fasa University of Medical Sciences, Fasa, Iran; 4 Department of Epidemiology and Biostatistics, School of Public Health, Imperial College London, London, UK; 5 Department of Nutrition, Bjørknes University College, Oslo, Norway; 6 Research Center for Social Determinants of Health, Jahrom University of Medical Sciences, Jahrom, Iran

**Correction: BMC Med Res Methodol 22, 305 (2022)**

**https://doi.org/10.1186/s12874-022-01791-7**

Following publication of the original article [1], the authors reported a mistake in the corresponding author’s affiliation, Fatemeh Rezaei. The corresponding author’s affiliation is: Research Center for Social Determinants of Health, Jahrom University of Medical Sciences, Jahrom, Iran

The original article has been updated.
